# Streamlined and Robust Stage-Specific Profiling of Gametocytocidal Compounds Against *Plasmodium falciparum*

**DOI:** 10.3389/fcimb.2022.926460

**Published:** 2022-06-30

**Authors:** Janette Reader, Mariette E. van der Watt, Lyn-Marié Birkholtz

**Affiliations:** ^1^ Department of Biochemistry, Genetics and Microbiology, University of Pretoria, Pretoria, South Africa; ^2^ Institute for Sustainable Malaria Control, School of Health Systems and Public Health, University of Pretoria, Pretoria, South Africa

**Keywords:** gametocyte, luciferase, malaria, *Plasmodium*, transmission-blocking, drug discovery

## Abstract

Malaria elimination is dependent on the ability to target both the pathogenic and transmissible stages of the human malaria parasite, *Plasmodium falciparum*. These forms of the parasite are differentiated by unique developmental stages, each with their own biological mechanisms and processes. These individual stages therefore also respond differently to inhibitory compounds, and this complicates the discovery of multistage active antimalarial agents. The search for compounds with transmission-blocking activity has focused on screening for activity on mature gametocytes, with only limited descriptions available for the activity of such compounds on immature stage gametocytes. This therefore poses a gap in the profiling of antimalarial agents for pan-reactive, multistage activity to antimalarial leads. Here, we optimized an effective and robust strategy for the simple and cost-effective description of the stage-specific action of gametocytocidal antimalarial compounds.

## Introduction

Increased resistance to currently available antimalarials continues to pose a threat to worldwide malaria elimination efforts and motivates the continued discovery of new antimalarial agents (World Health Organisation, 2020). Target profiling of candidate antimalarial agents now routinely requires evidenced killing of multiple life cycle stages of malaria parasites (Burrows et al., 2017). As such, antimalarial lead candidates are firstly profiled for activity against asexual blood stage (ABS) parasites, addressing the therapeutic necessity of killing parasite stages associated with morbidity and mortality. However, to be useful in and contribute to malaria elimination strategies, antimalarial candidates with such established ABS activity are typically profiled for additional abilities, including being able to block human-to-mosquito transmission of the parasite. This would thereby curb the spread of the disease, with various strategies proposed to exploit such transmission-blocking agents (Birkholtz et al., 2022).

In the human malaria parasite, *Plasmodium falciparum*, human-to-mosquito parasite transmission is dependent on the differentiation and development of mature gametocyte forms of the parasite. In this species, gametocyte development is a uniquely prolonged process (~12 days) characterized by five morphologically distinct stages (I–V) (Young et al., 2005), each of which is associated with distinct biological processes (Sinden, 2015; Ngotho et al., 2019; Van der Watt et al., 2022). After the stochastic conversion of ≤10% of *P. falciparum* ABS parasites to gametocytogenesis, immature gametocytes (stages I–IV) develop and sequester in extravascular environments including the bone marrow (Day et al., 1998; Aguilar et al., 2014). Terminally differentiated, mature falciform gametocytes (stage V) can re-enter the circulatory system and are the only gametocyte stage that can be transmitted back to the mosquito (Sinden, 2015; Meibalan and Marti, 2017; Ngotho et al., 2019). Mature gametocytes can be maintained in circulation for up to 55 days [mean life span, ~5.5 days (Bousema et al., 2010)] and are thereby continuously able to ensure transmission of the parasite in the human population, with only ~10^3^ mature gametocytes required for transmission to occur. Therefore, blocking human-to-mosquito parasite transmission is conceptually associated with the ease of targeting such low numbers of long-lasting mature gametocytes. Additionally, as they are circulating in the blood compartment, they are accessible to pharmacological intervention (Birkholtz et al., 2022).

Profiling of antimalarial candidates with ABS activity for additional transmission-blocking activity is frequently only performed on mature stage V gametocytes (D'alessandro et al., 2016; Plouffe et al., 2016; Van Voorhis et al., 2016; Miguel-Blanco et al., 2017; Paquet et al., 2017; Van Der Watt et al., 2018) or male and female gametes (Miguel-Blanco et al., 2017; Delves et al., 2018; Delves et al., 2019). In both instances, a reduction of oocyst numbers (and by implication sporozoite formation) is used to validate transmission-blocking activity (Vos et al., 2015; Birkholtz et al., 2016). However, such a screening pipeline would be fragmented and would not consider the different gametocyte stages of *P. falciparum*, each stage of which is associated with differential biological activities (Delves, 2012; Meibalan and Marti, 2017; Ngotho et al., 2019; Schneider and Reece, 2021; Usui and Williamson, 2021). This translates to compounds having variant activity profiles against these different stages of *P. falciparum* gametocytes (Duffy and Avery, 2013; Miguel-Blanco et al., 2017; Delves et al., 2018; Delves et al., 2019; Abraham et al., 2020). Although the majority of ABS actives do retain activity against immature gametocytes, very few are active against mature gametocytes (Duffy and Avery, 2013). Importantly, not all ABS actives are able to target immature gametocytes. For example, atovaquone, which is potent against ABS parasites, has no activity against immature and mature gametocytes (Plouffe et al., 2016). In these instances, if the entire ABS population is not rapidly cleared, any gametocytes that are formed and not targeted in their immature stages will continuously seed formation of mature gametocytes that will then be transmitted. In such scenarios, transmission will not be blocked, and the parasite will continue to be spread. Moreover, this is of particular concern given the presence of same cycle sexual conversion, i.e., induction of gametocytogenesis within the first cycle of ABS proliferation (Bancells et al., 2019) and evidence that some ABS actives (chloroquine, antifolates, and mefloquine) can shift the parasite population into sexual differentiation and increase the gametocytaemia of immature stages *in vivo* (Buckling et al., 1999; Price et al., 1999; Sowunmi and Fateye, 2003; Sowunmi et al., 2006; Fehintola et al., 2012; Thommen et al., 2022).

Evaluation of the stage-specific gametocytocidal activity of small molecules has been sparse (Duffy and Avery, 2013; Miguel-Blanco et al., 2017; Delves et al., 2018; Delves et al., 2019; Abraham et al., 2020) and, where these assays have been performed, they made use of extensive, complicated, and expensive manipulation of the gametocyte populations to enrich for specific stages (Adjalley et al., 2011; Lucantoni et al., 2013; Lucantoni et al., 2016; Plouffe et al., 2016). These processes run the risk of compromising gametocyte viability and negate the use of such assays in resource-constrained settings and for robust and reproducible high-throughput applications. Consequently, a more streamlined method is required to allow the stage-specific production of gametocytes with minimal manipulation, to accurately assess the activity of small molecules against immature and mature gametocyte populations. Additionally, the application of such an optimized, streamlined “whole-gametocytocidal” assay beyond proof of principle is required (Lucantoni et al., 2016). Here, we report an optimization of such whole-gametocytocidal assay to allow straightforward, robust, and routine investigation of stage-specific gametocytocidal activity of antimalarial candidates. We validate this strategy with key antimalarial lead candidates with known ABS activity and confirm the importance of characterising any new antimalarial lead compound for stage-specific action against various gametocyte stages including immature gametocytes. With this, we complete the gap in the current profiling of antimalarial candidates with potential transmission-blocking ability by including evaluation of stage-specific gametocytocidal activity.

## Materials and Methods

### Ethics Statement

Parasitology work and volunteer human blood donation at the University of Pretoria are covered under ethical approval from the Health Sciences Ethics Committee (506/2018) and Natural and Agricultural Sciences Ethics Committee (180000094).

### *In Vitro* Parasite Culturing

NF54-*Pfs16*-GFP-luc *P. falciparum* asexual parasites [kind gift from David Fidock, Columbia University, USA (Adjalley et al., 2011)] were maintained at 37°C in A^+^/O^+^ human erythrocytes at 5% hematocrit in complete culture medium under hypoxic conditions as previously described (Reader et al., 2015). Asexual parasites were synchronized at least twice at 12 h intervals with 5% (w/v) d-sorbitol. This staggered synchronization resulted in a parasite population containing >97% ring-stage parasites that were all detected and quantified morphologically to be within a tight developmental window of 5- to 10-h post-invasion. Such age binning and compartmentalization of these ring-stage parasites were performed as described before (Van Biljon et al., 2018; Van Biljon et al., 2019). All cultures were maintained with daily medium changes and synchronicity evaluated with Giemsa-stained thin smear stage binning under 1,000× magnification.

### Stage-Specific Gametocyte Production

**Figure 1:**
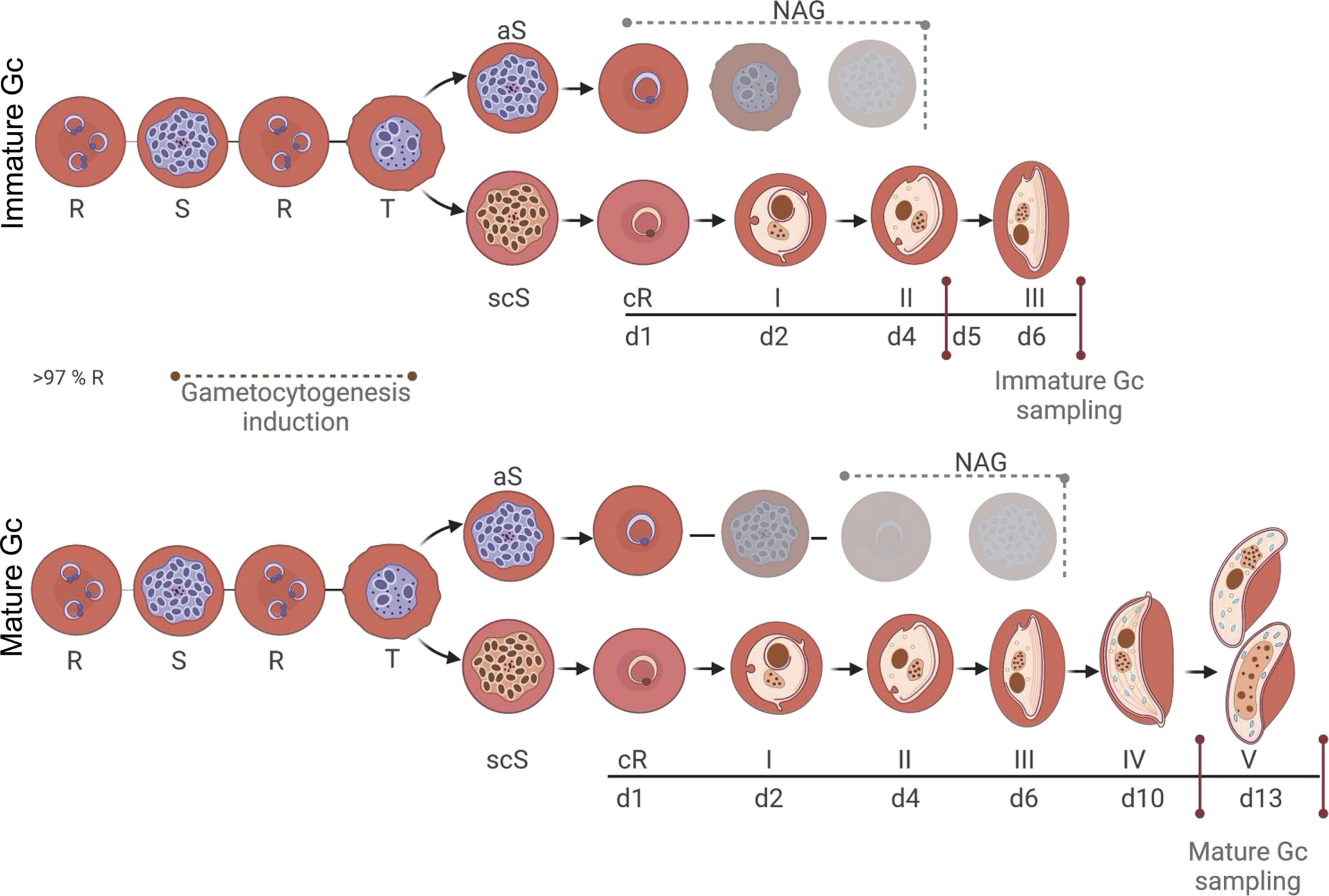
*P. falciparum* stage-specific gametocyte production and development. Asexual parasites (0.5% parasitemia, 6% hematocrit) were d-sorbitol synchronized for two consecutive cycles to ensure a highly synchronized (>97%) ring population. Initiation of gametocytogenesis (same cycle conversion) was induced on day 0 by a decrease in hematocrit, mimicking anemic conditions. Asexual parasites were eliminated with *N*-acetyl glucosamine treatment from days 1 to 4 for immature gametocytes and days 3 to 7 for mature gametocytes. Assays were performed on days 5–6 or 13 for immature and mature gametocytes, respectively. R-ring, S-schizont, T-trophozoite, aS-asexual schizont, scS-sexual committed schizont, cR-committed ring, Gc-gametocyte. Created and modified with BioRender.com.

Gametocytogenesis was induced on a tightly synchronised (>97% rings) asexual parasite culture (0.5% parasitemia and 6% hematocrit) with a combination of nutrient starvation and a decrease in hematocrit, as previously described (Reader et al., 2015). Gametocyte development was allowed to occur in culture medium prepared as for growth of asexual parasites but without additional glucose supplementation (day −3) under hypoxic conditions, stationary growth, with daily media replacement (from day −1) and parasites/gametocytes were morphologically monitored as per recent molecular descriptors (Brancucci et al., 2018; Dixon and Tilley, 2021). The hematocrit was reduced to 4% after 72 h (day 0), mimicking anemic conditions in the patient. For immature gametocytes (stage II/III), cultures were exposed to 50 mM *N*-acetyl glucosamine (NAG) on days 1–4 to eliminate residual asexual parasites and harvested at days 5–6 (**Figure 1**). For mature (stage V) gametocytes, NAG treatment only occurred from days 3–7 and harvested at day 13 (**Figure 1**).

### Male Gamete Formation Assay

The male exflagellation inhibition assay (EIA) was performed as described previously (Coetzee et al., 2020; Reader et al., 2021) by capturing the temporal movement of exflagellation centers by video microscopy. Mature *Pf*NF54 gametocytes (>95% stage V) were treated with 2 µM compound [with methylene blue (MB) as inhibition control at 10 µM] in complete culture media [final dimethyl sulfoxide (DMSO) concentration of <0.1% (v/v), 50% (v/v) A+ male human serum] for 48 h at 37°C under hypoxic conditions. Male gametogenesis was induced on these populations with 100 µM xanthurenic acid at room temperature for 16 min and exflagellation was measured in a Neubauer chamber. Movement was recorded by video microscopy (Carl Zeiss NT 6V/10 W Stab microscope, MicroCapture camera, 10× magnification) and quantified semiautomatically on 16 videos of 8–10 s each, captured between 16 and 24 min after incubation at 30 s intervals. The total exflagellating centers per treatment were quantified using ICY (open-source imaging software GPLv3) normalized to an untreated control.

### Viability Determination With Hydroethidine or Determining Adenosine Triphosphate Levels

The viability of both immature and mature gametocytes was evaluated microscopically by staining treated (DHA, 1× IC_50_ which equates to 20 nM and 5 µM on immature and mature gametocyte, respectively) and untreated gametocytes with 5 µM hydroethidine (HE) for 2 h at room temperature. After removal of excess dye with 1× PBS washes, parasites were fixed in 0.025% (v/v) paraformaldehyde onto a poly-l-lysine–coated cover slide, overnight at 4°C. Micrographs were generated with a Zeiss 510-Meta confocal laser scanning microscope and a HeNe laser with a 543 nm wavelength to excite HE.

Viability was also confirmed by determining ATP levels as a proxy for metabolic activity as previously described (Reader et al., 2015; Reader et al., 2021). Mature stage gametocytes (>90% stage V) were enriched using density gradient centrifugation in NycoPrep (Axis Shield) and magnetic separation using LS columns and a MidiMacs magnet. Approximately 62,000 gametocytes, in glucose-supplemented complete medium, were added to the well in a final volume of 100 µl. Plates were incubated for periods for 0, 12, 24, or 48 h in a humidified gas chamber (90% N_2_, 5% O_2_, and 5% CO_2_) at 37°C. Subsequently, the BacTiter-Glo™ assay (Promega) was performed according to the manufacturer’s instructions at room temperature in the dark, with assay substrate incubated for 10 min, to detect ATP levels. Bioluminescence was detected at an integration constant of 0.5 s with the GloMax^®^-Multi Detection System with Instinct^®^ software. MB (20 μM) was included as control.

### Stage-Specific Gametocytocidal Assay

Compounds [supplied by the Medicine for Malaria Venture (www.mmv.org), in DMSO] were evaluated after 48 h drug pressure on either immature (stage II/III) or mature (stage V) gametocytes (2% gametocytemia, 1.5% hematocrit) under hypoxic conditions at 37°C. Drug pressure of 48 h allowed standardization and comparison of activity of compounds if measured on additional transmission-blocking assays such as gamete and oocyst formation (Almela et al., 2015; Miguel-Blanco et al., 2017; Coetzee et al., 2020; Reader et al., 2021). Luciferase activity was determined in 30 μl of parasite lysate by adding 30 μl of luciferin substrate (Promega Luciferase Assay System) at room temperature and detection of bioluminescence at an integration constant of 10 s with a GloMax^®^-Multi Detection System with Instinct^®^ software. All single point screens were performed at 5 µM of the compound. All dose-response analyses were performed for 9–18 concentrations of 2-fold dilutions to determine the inhibitory concentration at which 50% of the gametocyte population was no longer viable (IC_50_). In both these assays, MB and the *P. falciparum* phosphatidylinositol-4-kinase inhibitor, MMV390048, were used as internal controls (at 5 µM each). Inhibition was enumerated as luciferase levels compared to control viable gametocytes and the IC_50_ with non-linear curve fitting and four parametric equations (GraphPad Prism). All assays were performed on three independent biological repeats, each in technical triplicates.

### Genotyping of the *Pf*NF54 Reporter Line

ABS parasites obtained from the NF54-*Pfs16*-GFP-luc reporter line expressing green fluorescent protein luciferase (Adjalley et al., 2011) were harvested and genomic DNA was isolated using Quick-DNA™ MiniPrep kit (ZYMO, Research, USA) according to the manufacturer’s instructions. Correct integration of reporter construct into the *cg6* locus was analyzed by PCR amplification of the fragments on each side of the attB integration site. The PCR fragments were amplified using KAPA Taq Ready Mix (Roche).

## Results

### Streamlined Production of Stage-Stratified, Viable Gametocytes

An essential requirement for the stage-specific evaluation of gametocytocidal activity of new antimalarial compounds is the compartmentalized production of immature (stage II/III) and mature (stage V) gametocytes (**Figure 1**). Our protocol relied on the use of highly synchronized ring-stage asexual parasites as a starting population (97.1 ± 0.6% synchronicity, all with <6-h age window, n = 50), from which gametocytes were induced (**Figure 1**). This allowed manipulation of the asexual population, which is less prone to adverse effects due to this treatment, rather than manipulation of the gametocyte population. For instance, magnetic purification of gametocytes, as is typically used in other protocols (Lelievre et al., 2012; Lucantoni et al., 2013; Lucantoni et al., 2016; Plouffe et al., 2016), results in a significant >2-fold drop in viability (*P* = 0.0004, paired, two-tailed *t*-test, n = 3) as rapidly as 12-h post-enrichment on stage IV/V gametocytes but is more pronounced at 24 and 48 h (*P* < 000.1, paired, two-tailed *t*-test, n = 3) (**Figure S1**). Importantly, to further ensure pure gametocyte populations, asexual parasites were continuously eliminated from the gametocyte population after induction at optimized exposure times for either immature or mature gametocyte populations (**Figure 1**), with <1 and 0.2% asexual parasites remaining in the immature (on days 5–6) or mature (day 13) gametocytes, respectively.

**Figure 2:**
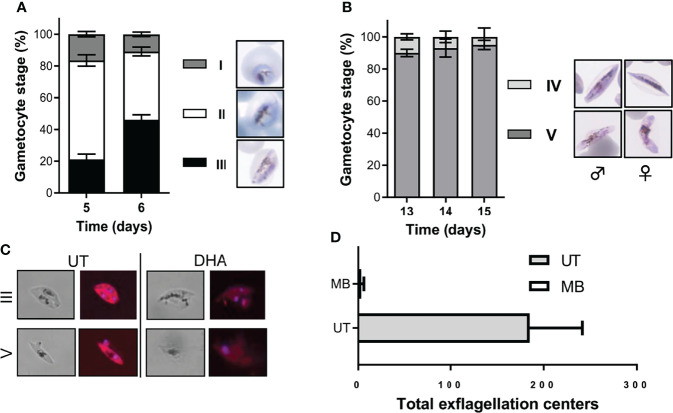
Morphological stage distribution and viability confirmation of immature and mature gametocytes. Quantification of the stage distribution of **(A)** immature gametocytes indicated at >80% enrichment to stage II/III gametocytes and **(B)** >90% stage V mature gametocytes on the day of assay. N > 30 individual exp, ~500 red blood cell counted/exp ± S.E. **(C)** Hydroethidine-stained confocal images confirmed the viability of untreated gametocytes and the “dead” phenotype observed following DHA treatment on both immature and mature gametocytes (at 1× IC_50_ of 20 nM and 5 µM, respectively). Magnification, ×1,000. **(D)** Viability of mature gametocytes was further evaluated by confirming male exflagellation before initiating any assays (N > 25, ± S.E.). MB, methylene blue (10 µM) treated gametocytes; UT, untreated gametocytes.

Immature gametocytes were harvested only when stringent morphological stage binning (**Figure S2**) indicated >80% enrichment to stage II/III gametocytes (**Figure 2A**; 16.5 ± 1.7% stage I, 62.3 ± 3.6% stage II, and 21.2 ± 3.3%, stage III; 826 gametocytes evaluated), with routine gametocytaemias of 3.3 ± 0.16% obtained (n = 81 independent inductions). These stage distributions were highly comparative to those obtained previously but with additional purification steps required (Lucantoni et al., 2013; Plouffe et al., 2016). Immature gametocyte populations containing any stage IV gametocytes were not used in downstream assays to ensure that drug efficacy was evaluated only on stage II/III gametocytes, even after 48-h drug pressure where the typical stage distribution remained at ~82% stage II/III gametocytes. By comparison, a more homogeneous population of mature stage gametocytes was obtained (2.7 ± 0.16% gametocytaemia, n = 50 independent inductions) consisting of >90% stage V gametocytes (10 ± 1.9% stage IV and 90 ± 2.3% stage V; 270 gametocytes counted; **Figure 2B**) due to continued removal of asexual parasites from day 3 and the natural attainment of endpoint differentiation (**Figure 1**). This stage distribution in the mature gametocyte population again correlates well with that obtained for other protocols, but without needing the additional purification as used in other protocols, e.g., through magnetic separation (Lucantoni et al., 2013; Plouffe et al., 2016), exposing committed rings to sorbitol treatment (Plouffe et al., 2016) or gradient separations (Lelievre et al., 2012), both during the gametocytogenesis process or downstream during the actual use of these gametocytes in gametocytocidal assays.

Stage-stratified gametocyte populations were produced here at low cost from a single parasite line and, in contrast to previous protocols, required minimal manipulation and no additional purification or enrichment steps. Our gametocyte populations were viable as measured with live-dead staining (**Figure 2C**) without any detectable morphological abnormalities. Additionally, mature gametocytes produced in this manner were fully functional and able to produce high numbers of exflagellating male gametes (15–20 exflagellation centers per field, 16 fields counted, average of 184.8 ± 56.7 total exflagellation centers per experiment; **Figure 2D**), comparable to previous work (13–50 exflagellation centers per field) (Delves et al., 2013).

### Evaluating Gametocytocidal Action on Different Stages of Gametocytes

**Figure 3:**
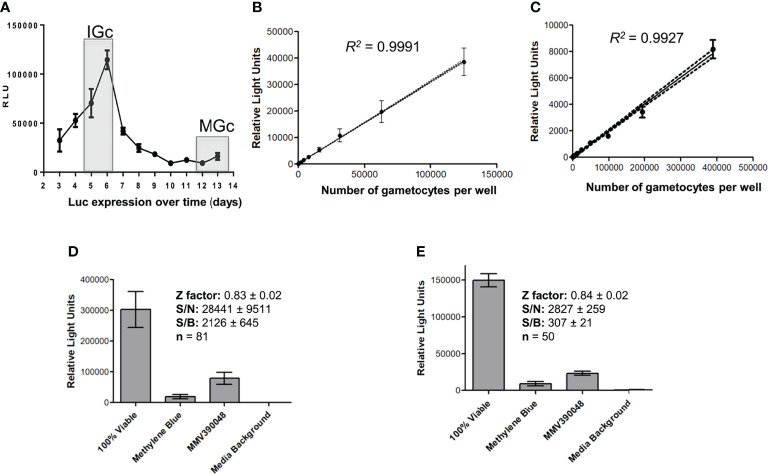
Luciferase reporter assay metrics. **(A)** Assessment of luciferase expression (RLU) throughout gametocytogenesis for the transgenic line NF54-*pfs16*-GFP-Luc on immature gametocytes (IGc, days 5–6), and the mature gametocytes (days 12–14, MGc). The linearity of luminescence readout compared to number of gametocytes per well for both the **(B)** immature and the **(C)** mature gametocytes. Data are from three independent biological experiments each performed in technical triplicates, ± SEM, 95% confidence intervals on each point indicated as ribbons. Assay metrics, including signal-to-noise (S/N), signal-to-background (S/B), and Z′-factor values for the luminescence readout (relative light units) on both the **(D)** immature and the **(E)** mature gametocyte assays, with 5 µM of either MMV390048 or MB.

The immature and mature gametocyte populations produced above were subsequently used to evaluate gametocytocidal action of compounds by detecting differences in luciferase signal after a 48 h incubation period. This signal remained detectable in the NF54-*Pfs*16-GFP-luc line used to produce the different stages of gametocytes for more than 10 generations (**Figure S3**). Luciferase expression could be detected throughout gametocytogenesis (**Figure 3A**) but was expectedly higher in immature gametocytes compared to mature gametocytes, as this is associated with *Pfs16* promotor activity that is higher in the immature stages but still active in mature stages (Bruce et al., 1994; Van Biljon et al., 2019). For both the immature and mature gametocytes, a linear relationship between gametocyte numbers and luciferase expression was present (*R^2^* [immature] = 0.9991 and *R^2^* [mature] = 0.9927; **Figures 3B, C**) with sensitive detection of signal in as little as ~3,000 gametocytes per well (in a 96-well format) for both immature and mature gametocytes. Saturation in the luciferase readout could not be observed at even the highest cell count tested (15 × 10^3^ immature gametocytes and ~50 × 10^3^ mature gametocytes per well), similar to previous reports with luciferase expression (D'alessandro et al., 2016). Overall assay performance indicated high reproducibility with Z′-factors of 0.83 ± 0.02 (immature gametocyte assay; n = 81) and 0.84 ± 0.02 (mature gametocyte assay, n = 50) routinely obtained (**Figures 3D, E**).

**Figure 4:**
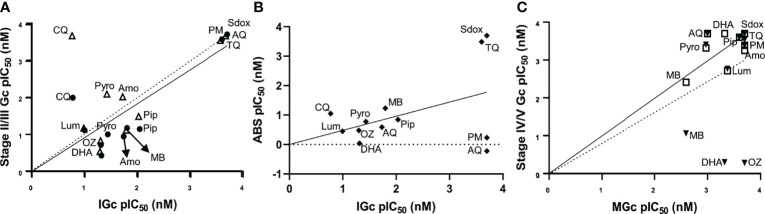
Stage-specific gametocytocidal action evaluation with known antimalarials. Correlation of the gametocytocidal activities of known antimalarials between the luciferase assay on **(A)** immature gametocytes and data from stage II (filled black circle; solid line) and stage III gametocytes (open triangle; dashed line) from a previous report (Plouffe et al., 2016). **(B)** Comparison between activity on immature gametocytes and ABS parasites. **(C)** Mature gametocyte activity compared to stage V (squares; solid line) and stage IV gametocytes (filled black triangles; dashed line) from a previous report (Plouffe et al., 2016). Data are from three independent biological experiments (n = 3), each performed in technical triplicates. DHA, dihydroartemisinin; OZ, artefenomel (OZ439); CQ, chloroquine; Amo, amodiaquine; Pip, piperaquine; Pyro, pyronaridine; Lum, lumefantrine; MB, methylene blue; AQ, atovaquone; PM, pyrimethamine; Sdox, sulfadoxine; TQ, tafenoquine.

To benchmark and validate the use of the stage-stratified gametocytes produced in gametocytocidal assays, a set of 12 known antimalarial compounds were evaluated against both immature and mature gametocytes and compared to published data (**Table S1**). The gametocytocidal activity of the compounds against our immature gametocyte population strongly correlated with that previously observed for both stage II (Pearson *R^2^* = 0.98) and stage III gametocytes (Pearson *R^2^* = 0.82; **Figure 4A**) (Plouffe et al., 2016) supporting the prevalence of stage II/III gametocytes in our immature gametocyte population and the absence of stage IV gametocytes (Pearson *R^2^* = 0.48 with stage IV gametocytocidal action as reported (Plouffe et al., 2016)). Moreover, the gametocytocidal action of the compounds against immature gametocytes correlated weakly (Pearson *R^2^* = 0.55; **Figure 4B**) with their activity against ABS parasites. Particularly, pyrimethamine and atovaquone that are potent ABS actives have no (>5,000 nM) activity against immature gametocytes (D'alessandro et al., 2016; Plouffe et al., 2016). The gametocytocidal action of the compounds against the mature gametocyte population confirmed the enrichment of stage V gametocytes, with a stronger correlation (Pearson *R^2^* = 0.82) with the stage V population from the “Plouffe” data set (Plouffe et al., 2016), compared to the stage IV population (Pearson *R^2^* = 0.48; **Figure 4C**). Comparison of individual IC_50_ values validated our data compared to previous reports [**Table S1**, (Lucantoni et al., 2013; Lucantoni et al., 2016; Plouffe et al., 2016)] and included, for instance, comparable IC_50_s of 20 and 0.9–7 nM for DHA on immature gametocytes (**Table S1**), while our mature assay accurately indicated the loss of activity of this compound on mature gametocytes (>2,000 nM), similar to data from Plouffe et al. (Plouffe et al. (2016)) and more accurate than those reported on late-stage gametocytes (Lucantoni et al., 2016; Coertzen et al., 2018).

**Figure 5:**
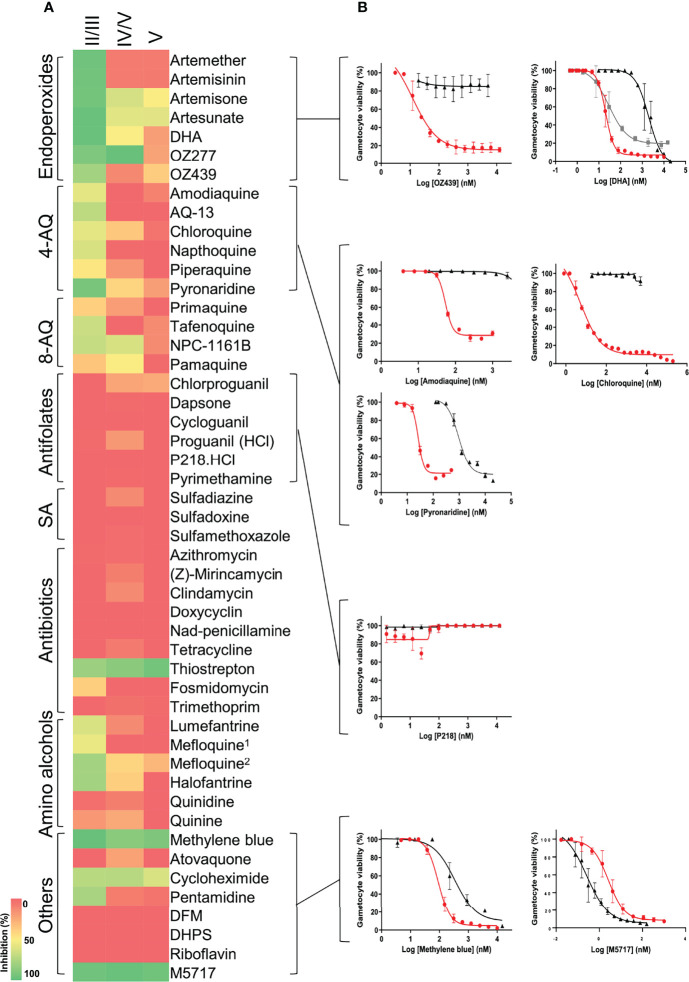
Evaluation of gametocytocidal activity of key MMV compounds. **(A)** Gametocytocidal profiling of the compounds (5 µM for 48 h) against immature (stage II/III), a mixture of stage IV and V and mature (stage V) gametocytes. **(B)** Dose-response curves of selected compounds indicating the IC_50_ shift between immature (red), mature (black), and mixed stage IV and V gametocyte populations (gray). ^1^Racemic, ^2^(+RS); DFM, deferoxamine mesylate salt; DHPS, dehydroepiandrosterone sulphate. Data are from three independent biological repeats (n = 3), performed in technical triplicates, mean ± S.E indicated.

When the stage-specific evaluation of gametocytocidal activity was extended to include additional investigative and frontrunner reference compounds from the Medicines for Malaria Venture (https://www.mmv.org/), we could confirm the presence of unique profiles associated with the susceptibility of different stages of gametocytes for different chemical classes (**Figure 5**). Amino alcohols (e.g., lumefantrine, halofantrine, and mefloquine), 4-aminoquinolines (such as amodiaquine, chloroquine, piperaquine, and pyronaridine), and the endoperoxides, which are all active against ABS parasites in a nanomolar range (Duffy and Avery, 2013), retain comparative activity in immature gametocytes with an average loss in activity of only ~3-fold observed for these classes. These compounds are inactive against mature stage V gametocytes (IC_50_ > 5 µM). The 8-aminoquinolines such as NPC-1161B and pamaquine show little activity on ABS (Duffy and Avery, 2013), which is also observed here (~60% inhibition for NPC-1161B and <50% inhibition for pamaquine at 5 µM; **Figure 5**), but the inactivity of primaquine in these *in vitro* assays is associated with its requirement of metabolic activation *in vivo*, as primaquine has validated transmission-blocking activity (Goncalves et al., 2016). Some activity for the above compounds is only reported when stage IV gametocytes were present (e.g., DHA with an IC_50_ of 88 nM in a stage IV/V population, but not active against stage V gametocytes; **Figure 5**), due to their known ability of targeting haem detoxification in the food vacuole, a process only active until stages III–IV of development and not important in mature, differentiated gametocytes (Lucantoni et al., 2013; Lucantoni et al., 2016; Plouffe et al., 2016). Only derivatives such as artemisone, artesunate, and artefenomel (OZ439) have some activity against mature gametocytes, associated with general disruption of redox homeostasis, as these processes are known to be downregulated in the mature gametocytes, enhancing the effect of increased reactive oxygen species production (Coertzen et al., 2018). Pyronaridine displays submicromolar activity against mature gametocytes (IC_50_ = 938 nM), in contrast to earlier reports (Duffy and Avery, 2013; Plouffe et al., 2016). The sulfonamides and antifolates (e.g., P218) have no activity against any stage of gametocytes, although antifolates are potently active against ABS (Duffy and Avery, 2013; Pethrak et al., 2022). The antibiotic, thiostrepton, retains activity against all stages, possibly through targeting general processes such as protein degradation. Importantly, more specific protein targets include the protein synthesis inhibitor with M5717 that inhibits elongation factor *Pf*EF2 (Baragana et al., 2015), equipotently active (IC_50s_ of 3.57 ± 0.7 and 0.25 ± 0.09 nM), against immature and mature gametocytes, respectively (*P* = 0.04, unpaired *t*-test, n = 3). This confirms its importance as frontrunner antimalarial candidate with ABS and gametocytocidal activity and its potential use in transmission-blocking strategies.

## Discussion

The recent detailed descriptors of the differential biology associated with the different stages of gametocytogenesis in *P. falciparum* have provided biological support and clarification for the stage-specific action of antimalarial compounds. It has become clear that profiling of compounds on immature gametocytes, in addition to mature gametocytes, is therefore of importance to progress leads and frontrunner clinical candidates as multistage active compounds. Such activities will ensure that sexual stages are targeted from the onset of gametocytogenesis and this will decrease the chances of mature gametocytes forming. In addition, if used in combination with an ABS active, but where the compound with gametocytocidal activity targets a different biological process, it will prolong the life span of ABS active as ABS-resistant parasites will not be able to spread (Birkholtz et al., 2022).

For stage-specific gametocyte assays to be routinely included to profile antimalarial candidates, we optimized a platform to allow robust, sensitive, and cost-efficient assays for stage-specific gametocytocidal activity to address the current caveat in the profiling of antimalarial candidates. This included the use of luciferase as viability indicator, produced from a single cell line and assayed in specific gametocyte stages, thereby proving previous proposals (Lucantoni et al., 2016) that such a whole-gametocytocidal assay is possible and can be widely applied to various antimalarial screening applications. Importantly, we prove that our platform allowed interrogation of the stage-specific gametocytocidal activity of frontrunner antimalarial candidates after 48 h drug pressure and therefore provide the first set of data to compare the activity of these compounds to data where they were evaluated on other life cycle stages after 48 h. Although longer incubation times (e.g., 72 h employed before) have improved compound activity against mature gametocytes (Lucantoni et al., 2017), this may indicate artificial potency as has been the case with DHA, where this compound could only be shown to be active on mature gametocytes upon longer incubation times (D'alessandro et al., 2016; Coertzen et al., 2018), inconsistent with the consensus that mature gametocytes are resistant to endoperoxides (Plouffe et al., 2016), as we confirm here. The reduced incubation period (compared to 72 h employed before) did not detrimentally affect assay performance, with high assay reproducibility still maintained (Z′-factors of 0.83 and 0.84 obtained on our platform with 48 h assay compared to previous reports of Z′-factors of 0.79–0.85 for a 72 h incubation on immature and mature gametocytes (Lucantoni et al., 2016). Additionally, using only a 48 h incubation on immature gametocytes ensures that stage-specific action is measured only on stage II/III gametocytes, while longer incubation periods run the risk of also measuring activity on developing stage IV gametocytes.

Additionally, our platform streamlined previous processes where either multiple *P. falciparum* luciferase expressing lines were required (Adjalley et al., 2011; Siciliano et al., 2017; Marin-Mogollon et al., 2019) or expensive instrumentation was needed, such as high-content imaging screening assays requiring expensive robotic imaging systems or monoclonal antibodies (Duffy and Avery, 2013; Lucantoni et al., 2015; Plouffe et al., 2016; Miguel-Blanco et al., 2017) rendering them impractical in developing countries or as routine profiling platforms. Similar to previous reports using the same cell line (Lucantoni et al., 2013; Lucantoni et al., 2016), the use of luciferase as viability indicator remains superior to other indicators including fluorescent signals (e.g., tdTomato), which may not be gametocyte- specific (Mclean et al., 2019) and requires expensive flow cytometry approaches (Matthews et al., 2020). Furthermore, luciferase is not influenced by assay differences as experienced with metabolic readouts (Reader et al., 2015; Reader et al., 2021). Importantly, expression of luciferase under the *pfs*16 promotor is gametocyte-specific and not influenced by the presence of asexual parasites (Lucantoni et al., 2013; Lucantoni et al., 2016). The latter is a major concern during drug screens particularly on immature gametocyte to distinguish activity of compounds from killing ABS parasites and therefore being seemingly active (Marin-Mogollon et al., 2019).

Previous stage-specific gametocyte assays made use of extensive, complicated manipulation of the gametocyte populations to enrich for specific stages (Adjalley et al., 2011; Lucantoni et al., 2013; Lucantoni et al., 2016; Plouffe et al., 2016), which compromises gametocyte viability and negates the use of such assays in resource constrained settings and in reproducible high-throughput applications. Furthermore, none of these methods result in any improved enrichment of specific stages above what we report here. Currently, stage stratification of gametocytes remains a mostly subjective process, dependent on expertise in morphological analyses of gametocyte stages. However, recent reports have guided this process as more detailed mechanistic data, and stage-specific features have been revealed (Dixon and Tilley, 2021), associated with the individual gametocyte stages in *P. falciparum.* Moreover, with the description of gametocyte-specific transcripts present exclusively in certain stages of gametocyte development (Van Biljon et al., 2019), these could be explored to generate lines with stage-specific expression of multiple reporter genes.

One of the main outcomes of this work is the unique gametocyte stage-specific profiles observed for an investigative and frontrunner reference set of compounds. This highlights the importance to investigate the chemosensitivity of the immature gametocytes to compounds in the developmental pipeline. Compounds that are unable to target immature gametocytes could result in seeding of mature gametocytes and could compromise their efficacy against these mature stages. The streamlined and robust process of gametocyte production and stage-specific evaluation of gametocytocidal activity of antimalarial compounds described here therefore provides affordable and sustainable enabling technology, which is immediately useful to studies performed in developing countries.

## Data Availability Statement

The original contributions presented in the study are included in the article/**Supplementary Material**. Further inquiries can be directed to the corresponding author.

## Author Contributions

L-MB conceived the study with JR and MvdW performing all assays and doing data analyses. JR and L-MB wrote the paper with input from MvdW. All authors contributed to the article and approved the submitted version.

## Funding

L-MB acknowledges the South African Medical Research Council Strategic Health Innovation Partnership and the Department of Science and Innovation South African Research Chairs Initiative, administered through the South African National Research Foundation (UID 84627). Research is supported by a BMGF Grand Challenges Africa grant (GCA/DD2/Round10/021/001) and by the Medicines for Malaria Venture as Global Test center for stage-specific gametocytocidal assays to L-MB. The UP ISMC acknowledges the South African Medical Research Council (SA MRC) as Collaborating Centre for Malaria Research.

## Conflict of Interest

The authors declare that the research was conducted in the absence of any commercial or financial relationships that could be construed as a potential conflict of interest.

## Publisher’s Note

All claims expressed in this article are solely those of the authors and do not necessarily represent those of their affiliated organizations, or those of the publisher, the editors and the reviewers. Any product that may be evaluated in this article, or claim that may be made by its manufacturer, is not guaranteed or endorsed by the publisher.
