# Supplementary material for: Streamlined and Robust Stage-Specific Profiling of Gametocytocidal Compounds Against Plasmodium falciparum
Source: Front Cell Infect Microbiol. 2022 Jun 30;12:926460. doi: 10.3389/fcimb.2022.926460 (PMC9282888; doi:10.3389/fcimb.2022.926460)
Supplement: Supplementary file 1 [file Image_1.pdf]

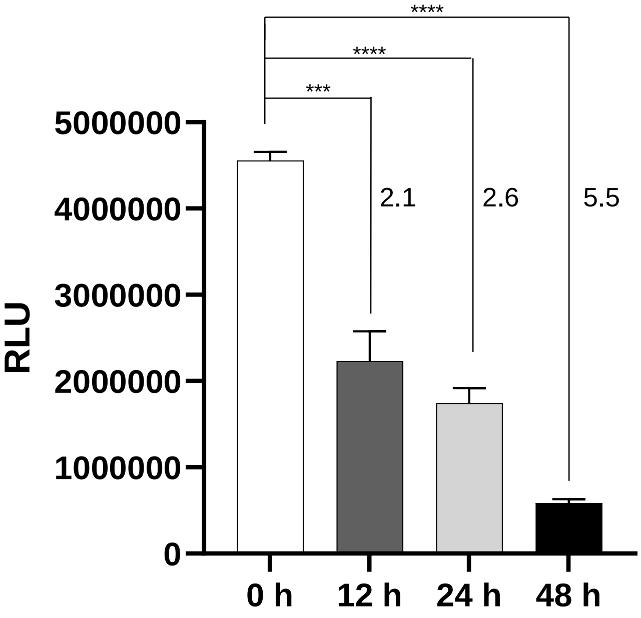

Figure S1. Measurement of relative light units (RLU) as a proxy for gametocyte viability, following magnetic separation as purification method without drug pressure. Following magnetic separation, the ATP production of the gametocytes were either read immediately (0 h, white bars) or incubated for 12 h (dark grey bars), 24 h (light grey bars) or 48 h (black bars) to determine the viability of the gametocytes. A decrease of 2.1 x (P = 0.0004, paired, two-tailed t test) was observed between 0 h and 12 h incubation, in comparison with a 2.6 and 5.5 x drop in viability following 24 h and 48 h ($P < 0.0001$, paired, two-tailed t test). Data are from three independent biological repeats (n = 3), performed in technical triplicates, mean ± S.E indicated.

I

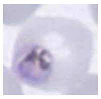

The earliest stage of gametocytes development is morphologically indistinguishable from the asexual ring stage.

Stage I gametocytes: similar to an asexual trophozoite, with differentiation due to the hemozoin crystals that do not form punctate clusters, as observed in asexual parasites.

IIa

IIb

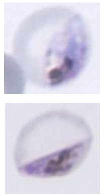

Stage II parasites have a visible granular distribution of pigment in several food vacuoles adopt a lemon shape with mononuclear content.
Size of the gametocyte is half of a normal red blood cell.

III ♂ ♀

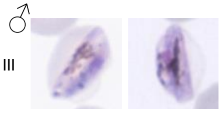

Stage III gametocyte's nucleus and mitochondria are extended (length to width ~2:1) and adopt a D-shape, due to the ultrastructural changes such as subpellicular membrane and adjacent microtubules.
The sex of the gametocytes can be morphologically determined for the first time when the parasite enters stage III
Morphologically, the male nucleus appears larger and the female cytoplasm contains more mitochondria.

IV

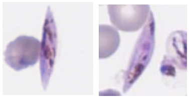

Stage IV: Both males and females are elongated and thin with sharp pointed ends, adapting the crescent banana shape (length to width ratio of 4:1).

V

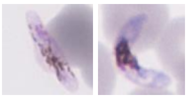

Stage V: Is the uniquely and characteristic falciform shape. The stage V male gametocytes are stouter and its cytoplasm appears pale blue following Giemsa staining, with reduces ribosome and ER network. Stage V female gametocytes are extended, and more curved with a bright Giemsa stained cytoplasm and concentrated nuclear material.

Figure S2. Detailed description of the morphology of the different sexual stages of the *P. falciparum* parasite. Descriptive morphological characterization of the five distinctive sexual stages used for the binning of stage distribution determinations as per recent molecular descriptions from Dixon and Tilley 2021; Brancucci *et al.* 2018..

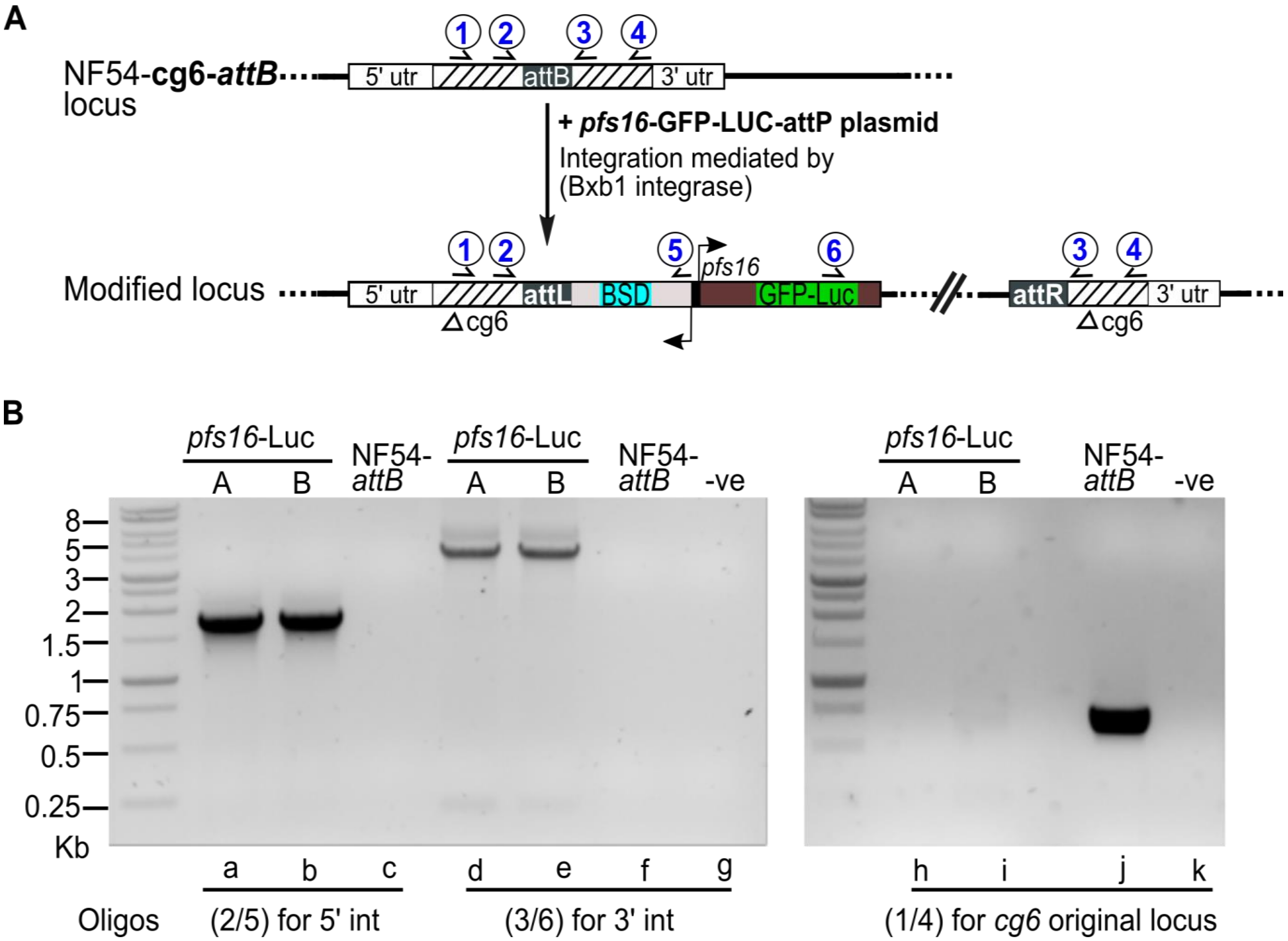

Figure S3. PCR validation of a *Pf*NF54 reporter line expressing GFP-luciferase (Adjalley *et al.,* 2011) under control of the gametocyte-specific promoter, *pfs16.* **(A)** Schematic of integrase-mediated *pfs16*-GFP-LUC-*attP* plasmid insertion into the *cg6-attB* locus; via single crossover recombination between incoming *attP* and chromosomal *attB* sites. This produced non-identical *attL* and *attR* sites that flank the integrated plasmid. **(B)** Diagnostic PCR confirming the correct 5' integration (lanes a-c, primers 1/2; 1.6 Kb), 3' integration (lanes d-g, primers 3/6; 4.5 Kb), and absence of the *cg6-attB* locus (lanes h-k, primers 1/4; 690 bp) in the GFP-luciferase reporter line (*pfs16*-Luc). DNA obtained from NF54-*cg6-attB* (NF54-*attB*) parental line served as control.
