# Supplementary material for: Streamlined and Robust Stage-Specific Profiling of Gametocytocidal Compounds Against Plasmodium falciparum
Source: Front Cell Infect Microbiol. 2022 Jun 30;12:926460. doi: 10.3389/fcimb.2022.926460 (PMC9282888; doi:10.3389/fcimb.2022.926460)
Supplement: Supplementary file 2 [file Table_1.pdf]

Table S1: Comparison of inhibitory activity of 12 known antimalarial drugs on immature and mature stages of the *P. falciparum* gametocytes as obtained from different assay platforms on different gametocyte stages.

Compound	IC_{50} (nM)							
	NF54^{Pfs16} luciferase (48 h)*		Mitotracker (SALSA)#				NF54^{Pfs16} luciferase (72 h)&,$	
	Immature	Mature	Stage II	Stage III	Stage IV	Stage V	Immature&	Late stage$
**DHA**	20.5	2103	2.7	6.7	2.1	>5000	0.9	91.5
**OZ439**	20	>5000	5.2	3.4	2	>5000	ND	ND
**Chloroquine**	5.92	>5000	98	>5000	>5000	>5000	76.75	ND
**Amodiaquine**	54	>5000	8.8	107	2460	1780	189.71	ND
**Piperaquine**	108	4000	14	31	4000	4000	ND	ND
**Pyronaridine**	27	938	10	126	2579	2075	168.09	4914.3
**Lumefantrine**	9.88	2382	13	15	599	520	7.82	ND
**Methylene Blue**	62.7	396	15	13	12	258	29.64	38.3
**Atovaquone**	>5000	1000	>5000	>5000	>5000	>5000	>10 000	ND
**Pyrimethamine**	>5000	>5000	>5000	>5000	>5000	>5000	> 40 000	ND
**Sulfadoxine**	>5000	>5000	>5000	>5000	>5000	>5000	ND	ND
**Tafenoquine**	3988	5000	3680	3730	3484	2449	3628	2505.1

* Data obtained from the assay platform developed here (48 h incubation)
# Data obtained from the SALSA assay in Plouffe et al. 2016 (72 h incubation)
& Data as published in Lucantoni et al. 2013 (72 h incubation)
$ Data as published in Lucantoni et al. 2016 (72 h incubation)
